# Nutrigonometry IV: Thales’ theorem to measure the rules of dietary compromise in animals

**DOI:** 10.1038/s41598-023-34722-7

**Published:** 2023-05-08

**Authors:** Juliano Morimoto

**Affiliations:** 1 Institute of Mathematics, University of Aberdeen, King’s College, Aberdeen, AB24 3FX Scotland; 2 School of Biological Sciences, University of Aberdeen, Zoology Building, Tillydrone Ave, Aberdeen, AB24 2TZ Scotland; 3 Programa de Pós-graduação em Ecologia e Conservação, Universidade Federal do Paraná, Curitiba, 82590-300 Brazil

**Keywords:** Behavioural ecology, Evolutionary ecology

## Abstract

Diet specialists and generalists face a common challenge: they must regulate the intake and balance of nutrients to achieve a target diet for optimum nutrition. When optimum nutrition is unattainable, organisms must cope with dietary imbalances and trade-off surplus and deficits of nutrients that ensue. Animals achieve this through compensatory rules that dictate how to cope with nutrient imbalances, known as ‘rules of compromise’. Understanding the patterns of the rules of compromise can provide invaluable insights into animal physiology and behaviour, and shed light into the evolution of diet specialisation. However, we lack an analytical method for quantitative comparisons of the rules of compromise within and between species. Here, I present a new analytical method that uses Thales’ theorem as foundation, and that enables fast comparisons of the rules of compromise within and between species. I then apply the method on three landmark datasets to show how the method enables us to gain insights into how animals with different diet specialisation cope with nutrient imbalances. The method opens new avenues of research to understand how animals cope with nutrient imbalances in comparative nutrition.

## Introduction

Evolution has shaped animals to integrate internal (physiological) and external (behavioural, social, ecological) cues to balance their nutrition^1,2^. Whether diet specialists or generalists, all animals must regulate the intake and balance of nutrients in their diet to maximise growth and fitness^3,4^. Despite this, optimum nutrition (henceforth referred to as the ‘intake target’) is not always attainable, and animals must cope with nutrient imbalances the best way possible. The rules that animals follow in order to compromise their nutrient intake remains subject of extensive interest in nutritional sciences research because they shape animal decision-making and long-term fitness^5^.

**Figure 1:**
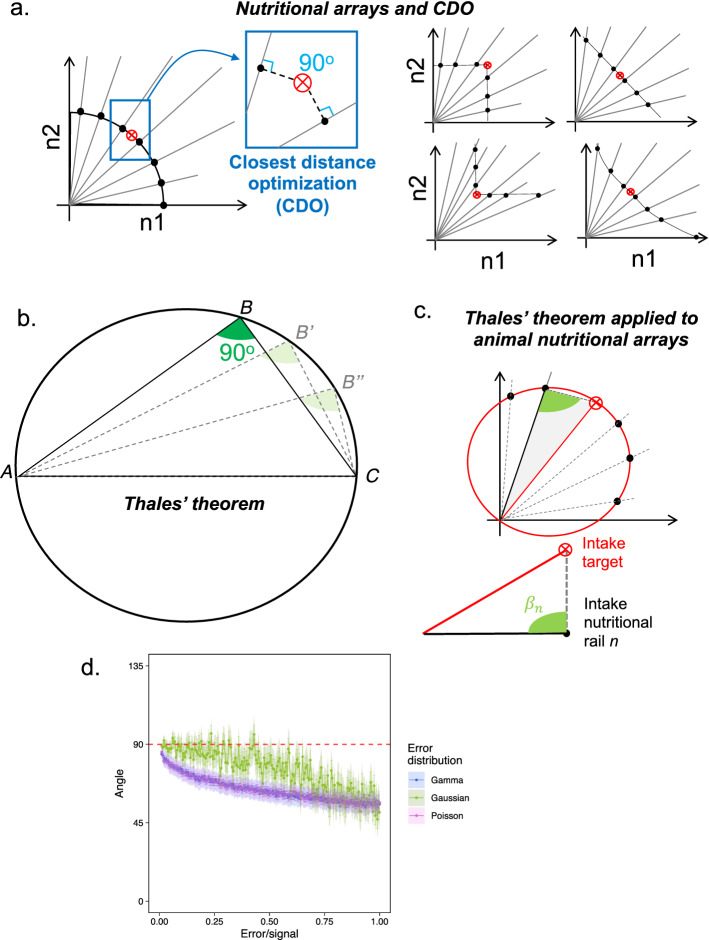
Nutritional arrays, closest distance optimisation (CDO), and the Thales’ theorem. (**a**) An example of a hypothetical GF nutritional array for the intake of nutrients *n*1 and *n*2. CDO is an array in which the distance between the intake in an imbalanced nutritional rails are minimised relative to the intake target (red crossed point). This implies that the angle between the nutritional rail intake and the intake target is 90° (see zoomed blue box). Nutritional arrays do not have to adopt CDO, and can have a wide range of configurations (left panels) such as a square array, equal distance array, inverted square array, and a concave array (read small panels clockwise). This is reviewed in details in^6,11^. (**b**) Thales’ theorem states that an inscribed triangle will have angle $\beta$ = 90° when the vertices lie on the circumference and the side ${\overline{AC}}$ is the diameter. Note the angle remains the same as long as these conditions are fulfilled (see faded triangles with vertices $\beta '$ and $\beta ''$ (**c**) One can apply Thales’ theorem to investigate whether or not nutritional arrays matches the predicted conditions for CDO, or how much and where the nutritional array deviates from CDO. (**d**) Normal distribution of errors provides more stability for the estimates of the angle $\beta$. X-axis represent the proportion of ‘noise’ (effect size over error and the y-axis is the estimate of the angle $\beta$ following the Thales approach. Shaded region represents the 95% confidence intervals of the simulations.

Animals can regulate nutrient imbalances physiologically, through post-digestive processes (see e.g.,^6–9^) and behaviourally, by choosing the amount and types of foods to eat and hence, controlling the magnitude of nutrient surpluses and deficits^3^ (see also^10^, for excellent review on the topic). Both of these processes are expected to follow rules (aka ‘rules of compromise’) that evolved under natural selection which enable animals to tolerate nutrient imbalances^3^. These rules of compromise aim to minimise the costs of surpluses and deficits of nutrient intake in imbalanced diets^11^. Marked differences in the rules of compromise have been described in closely related species with different dietary needs (e.g.,^8,10,12^) but this is a new field for which large-scale comparative studies remain a fertile field of investigation. A recent framework has enabled us to unravel such rules of compromise: the framework known as the Geometric Framework for nutrition (GF). GF accommodates the complexity of nutrition through a clever experimental design where the additive and interactive effects of nutrients can be investigated simultaneously^11,13^.Two concepts in the GF framework are key: *nutritional rails* and *intake target*. Nutritional rails are diets with fixed ratio of nutrients that animals are fed, and can be balanced or imbalanced. Animals can move along these nutritional rails by modulating the quantity of food they eat, but cannot move across rails because the ratio of nutrients is fixed (hence the name ‘rails’), unless a choice experiment is performed (see Fig. 1a). In standard GF experiments, the number of nutritional rails can vary, but is typically between five and ten (e.g.,^13–23^). These rails are essential to generate the nutrient array, which is the collection of average food intake of animals in each of the nutritional rails^11^ (Fig. 1a). The intake target is the balance of nutrients that animals *actively* seek to achieve when allowed to feed freely^6,11,13^. The intake target is the closest measure of the optimum nutrient balance of animals in terms of food consumption (although other targets may exist, e.g., for growth) (see^6,24^, for thorough discussion). The rules of compromise can thus be inferred from the way animals feed on the nutritional rails relative to their intake target (i.e. the shape of the nutrient array). This is precisely why nutrient arrays can be used as the fingerprint of the underlying rules of compromise guiding animal feeding^3^. Rules of compromise are generally assumed to impose constraints on how animals feed when the available diet differs (by a little or a lot) from the optimal diet. A more detailed overview of the GF framework can be found in the literature (see e.g.,^4,6,11,13,18,25,26^, and others). Importantly, GF is a framework that can be applied across taxa, making GF an attractive framework to reveal general patterns and responses in animal nutrition^11,13^. Because of this, GF has gained popularity in studies of nutritional ecology, particularly when the focus is on nutritional trade-offs and life-history traits (see e.g.,^10,14,18,22,23,27–30^, and references therein). Although GF experiments are expensive and time-consuming, and broader data sharing remains poor^25^, GF has enabled unprecedented insights into animal and human nutrition (see e.g.,^5,6,10,31–35^).

An important roadblock for the wider use of GF has been the relative delay in the development of specific analytical frameworks that match the experimental complexity of GF studies. Until recently, studies have relied at least partly on visual interpretations of multidimensional performance landscapes obtained with GF to draw biological conclusions (see e.g.,^15,27,36,37^) (but see also^28^), making objective comparisons and comparative studies of nutrition using GF difficult. Recent models have been developed to address this, and this has been a fertile ground for methodological advances (see for instance^18,26,28,38–42^). However, the same level of methodological development has not been seen for studies on the rules of compromise, which has lagged behind and remains in need of analytical breakthroughs. Analytical methods are crucial for the advancement of this field because they provide the methodology for accurate and reproducible analyses of the rules of compromise. This can help uncover insights into the eco-evolutionary processes underpinning diet specialisation. For example, diet specialists, but not diet generalists, display a peculiar rule of compromise known as the ‘closest distance optimisation’ (CDO)^11^ (see^6^, for a review) (see Fig. 1a), which was empirically observed in dietary specialist locust and moth species^8,12,43,44^ (see also^45^). Furthermore, CDO was observed in the solitary but not the gregarious stage of the swarming locust *Schistocerca gregaria*, suggesting that solitary individuals might have more specialised diets as opposed to the swarming gregarious counterparts^8^.

There have been few specific studies developing theoretical methods for quantitative analysis of the rules of compromise. For example,^11^ has provided conceptual overview of the nutrient intake arrays that animals display in GF studies, which can be used to infer the rules of compromise. Later,^3^ provided guidelines to study and interpret nutrient arrays and the associated rules of compromise. At the time, the proposed approach relied on Euclidean distances between the amount of food eaten between the imbalanced and optimal diets, which were plotted in 2D spaces to generate what is called ‘summary plots’ (e.g., Figs. 4 and 5 in^3^). However, summary plots estimate the Euclidean distances for each nutrient in the data separately, resulting in a plot with two (or more) curves with different patterns that can be challenging to interpret. In general, the shape of these curves has been interpreted individually and, depending on their linearity and non-linearity, inferences on the rules of animal compromise were derived^3^. This can be problematic due to some degree of subjectivity. A more complex model was later developed which involved the mapping of the nutrient arrays onto performance landscapes to compare the overall shape of the performance landscape relative to the shape of the nutrient arrays^46^. A geometric model was proposed by^47^ which also relied on Euclidean distances between points to find what was called the ‘regulatory scaling factor’, which estimated how organisms cope with nutrient surpluses and deficits^47^. These models also relied on the Euclidean distances between two average points (e.g., the average intake in imbalanced and the target diets) and lacked error estimates (see e.g.,^8,43,47^). Other models have been developed to analyse the trade-off between energy intake and the leverage that each nutrient has in shaping animal nutrient arrays, which have been applied to human nutrition to gain insights into obesity^48,49^. However, these past methods often relied on pairwise distances between average diet intakes in balanced (intake target) and imbalanced diets (nutritional rails). Thus, an integrative method that enables clear visualisation and rapid and intuitive computation of CDO will benefit our interpretation and understanding of animal feeding rules, advancing the field of nutritional ecology.

Here, I propose an analytical method to address this gap. The method builds upon the interpretation of distance summary plots from^3,46^ but enables direct statistical tests of the patterns of nutrient arrays against the predictions from CDO. This is achieved by integrating an ancient mathematical theorem known as the *Thales’ theorem* to estimate deviations of the patterns of nutrient arrays from CDO (see Fig. 1b,c), which I show here by validating the method to a simulation (Fig. 1d) and applying the method to three landmark datasets of increasing complexity: (1) the data for the nutrient array of a single species, *Drosophila melanogaster*, (2) the data for locusts presented in^8^, and (3) the data for generalists and specialists *Spodoptera* moths from^43,44^. I used these datasets of nutrient arrays that emerged from behavioural regulations of food intake (as opposed to post-digestive processes), but the method presented here is also applicable to physiological and molecular data from GF. Overall, the method proposed here advances our ability to make statistical inferences on the rules of compromises within and between species. This opens up new possibilities to study how the rules of compromise evolved across the animal kingdom, and how the behaviour, ecology and physiology of species can influence their ability to cope with nutrient imbalances.

## Results

### The method: Thales’ theorem and CDO

Thales’ theorem states that if an inscribed triangle has points *A*, *B* and *C* on the circumference, where the side ${\overline{AC}}$ is the diameter of the circumference, then the angle $\angle {ABC}$ equals to $\frac{\pi }{2}$ (i.e., 90°) (see Fig. 1b). But why is this theorem useful in the context of the rules of compromise?

A common and informative rule of compromise is the CDO (see ‘Introduction’), which states that animals should minimise the distance between the average intake of the imbalanced diet relative to the intake target. This leads to a semi-circle configuration of the nutrient array^3^ (see Fig. 1a). The CDO configuration emerges because in a flat plane, such as the Cartesian plane (or more generally, in ${\mathbb {R}}^2$) the closest distance between two points is a straight line. This means that the closest distance from the intake target and a point in a nutritional rail is a straight line with 90°angle between the rail that crosses the intake target and the imbalanced nutritional rail *i*, where *i* is the number of imbalanced nutritional rails used in the study^11^. Recall that the Thales’ theorem states that the angle $\beta = \angle {ABC}$ equals 90° if and only if the three points of an inscribing triangle lie in the circumference and ${\overline{AC}}$ is the diameter. Adapting this theorem, we can draw a circumference with diameter equal to the distance between the origin and the intake target. We can then triangulate the origin, the intake target, and the point in the imbalanced nutritional rail such that if the angle $\beta$ equals to 90°for all nutritional rails, then the nutrient array is that of a CDO rule of compromise (see Fig. 1c). Moreover, if the nutrient array does not match that of CDO rule of compromise, the angle $\beta$ can nevertheless provide useful insights to determine the relative importance of each nutrient in determining animals’ feeding priorities, such as e.g., which nutrients are more or less tightly prioritised. For example, if the angle $\beta$ is greater than 90°, then the point in the nutritional rail lies *inside* the inscribing circle, which suggests stronger feeding constrain to avoid surpluses of a nutrient. Conversely, if the angle $\beta$ is smaller than 90°, the point in the nutritional rail lies *outside* the inscribing circle, suggesting that the surplus of the nutrient is well tolerated. Interestingly, the simulations of error structure underpinning nutrient array data showed that the Thales approach to estimate the angle $\beta$ is more stable when the error distribution in the nutritional rails is derived from a Gaussian distribution (see Fig. 1d).

### Drosophila responds to dietary imbalances with underconsumption of carbohydrate but not of protein

**Figure 2:**
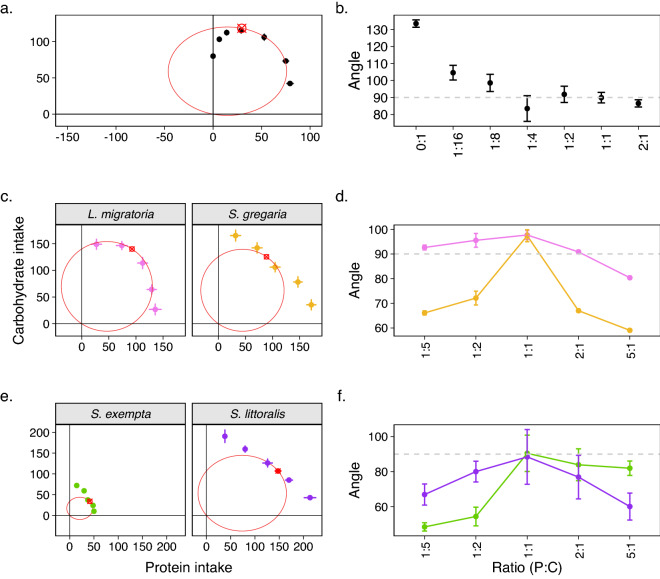
Thales’ theorem applied to empirical nutritional arrays (**a**) Nutritional array in *Drosophila melanogaster*, with mean diet intake for each imbalanced diets (with varying P:C ratios) from^14^. (**b**) Summary plot of the angle $\beta$ of the nutritional rails relative to the intake target. A 90° angle suggests that the nutritional array matches the prediction of CDO for a given rail. (**c**) Nutritional array in *L. migratoria* and *S. gregaria*, with mean diet intake for each imbalanced diets (with varying P:C ratios) extracted from^8^. (**d**) Summary plot of the angle $\beta$ of the nutritional rails relative to the intake target for the two species. (**e**) Nutritional array in *S. exempta* and *S. littoralis*, with mean diet intake for each imbalanced diets (with varying P:C ratios) extracted from^43,44^. (**b**) Summary plot of the angle $\beta$ of the nutritional rails relative to the intake target for the two species. Red circle: Thales’ circle with diameter equals to the intake target.

Firstly, I applied the method to gain insights into the feeding behaviour of *Drosophila melanogaster*. When given a choice, *Drosophila* regulates the intake of both protein and carbohydrate to reach a P:C ratio of 1:4, which is the ratio that maximises lifetime egg production (fitness)^14^. Moreover, when given a choice between two complementary diets of varying concentrations, flies also choose to overeat protein when the concentration of carbohydrate is low in the counterpart diet^14^. Using the method proposed here, I confirmed that flies are able to regulate both protein and carbohydrate intakes ($F_{7,966}$: 15.585, *p* < 0.001, Table 1) , as the shape of the nutrient array for diets with P:C ratio 1:4, 1:2, 1:1 and 2:1 were according to the predictions of the CDO (see Fig. 2a, b). However, as the nutrient imbalances in the diet increased towards high-carbohydrate contents (i.e., P:C 1:8, 1:16, 0:1), dietary intake of the nutritional rails progressively decreased, becoming statistically significantly different than the predictions from CDO for diets with P:C 1:16 and 0:1 (see Fig. 2a, b). These results suggest that flies display remarkable underconsumption of carbohydrate-biased, but not protein-biased diets when facing strong nutrient imbalances (Table 1).

**Table 1:** Estimates of angle $\beta$ in the nutritional array in *D. melanogaster* relative to CDO.

P:C ratio	*D. melanogaster*		
	Mean $\beta$	lwr 95% CI	upr 95% CI
0:1	43.526	34.643	52.410
1:16	14.596	5.676	23.508
1:8	8.587	−0.460	17.634
1:4	−6.503	−15.355	2.348
1:2	1.849	−6.908	10.607
1:1	−0.093	−9.041	8.854
2:1	−3.469	−12.258	5.320

Note that ratios in which the angle $\beta$ overlaps zero implies no differences from CDO.

### Diet specialisation leads to different nutrient arrays in response to dietary imbalances in two locust species

Next, I applied the method to the dataset of *Locusta* and *Schistocerca* locusts species first presented in^8^. The original study shows the difference in the nutrient array, whereby *Locusta*, a diet specialist, displayed nutrient array as predicted by CDO whereas *Schistocerca*, a diet generalist, displayed a more linear nutrient array which was more tolerant of overconsumption of both protein and carbohydrate (see Fig. 4 in^8^). The method presented here provides a clear framework to distinguish between the two responses (see Fig. 2c, d). The method corroborates the findings presented in^8^ by showing that the nutrient array for *Locusta* fits well to the predictions for CDO, while the response for *Schistocerca* diverged substantially. This translated into different patterns in the plots of the angle $\beta$ for both species. For instance, the angle $\beta$ for *Locusta* fluctuated closely to 90°  as expected from the Thales’ theorem predictions. Meanwhile, the angle $\beta$ for *Schistocerca* was often smaller than 90° in nutritional rails with more extreme nutrient imbalances, and progressively converged to 90° as the P:C ratio of the nutritional rail approximated the optimum P:C ratio of the intake target. This led to the plot of the angle $\beta$ to resemble a parabola (see Fig. 2d). Thus, the method proposed here provides an analytical framework to clearly differentiate differences in nutrient arrays and deviations from CDO.

### Nutrient-specific effects of diet specialisation levels on the responses to nutrient imbalances in *Spodoptera* species

Next, I used the method to gain insights into the patterns of the nutrient arrays of two *Spodoptera* species with different diet specialisation levels. There was substantial difference in the overall consumption of diets (irrespective of their P:C ratios) between the two species, with *S. littoralis* consuming greater amounts of all diets (see Fig. 2e, f). More importantly, the method revealed interesting differences in the responses to nutrient imbalances between the two species (see Fig. 2e). There was a statistically significant interaction between species and the nutritional rail on the estimates of the angle $\beta$ (Ratio * Species: $F_{4,626}$: 13.668, p < 0.001), suggesting that the shape of nutrient arrays differ between species.

*Spodoptera littoralis*, a diet generalist species, displayed a clear pattern of overconsumption of the most abundant nutrient in order to minimise the underconsumption of the least abundance nutrient. This was true for all four days of feeding data collection and resembled the responses of *Schistocerca* (see above). This means that, if we were to connect the average intakes across all nutritional rails to form the nutrient array of *S. littoralis* (or *Schistocerca* previously), forming a parabola on the plot of the angle $\beta$ (see Fig. 2e, Table 2).

**Table 2:** Estimates of angle $\beta$ in the nutritional array in locusts and moths, relative to CDO.

Species	P:C ratio	Mean $\beta$	lwr 95% CI	upr 95% CI
*L. migratoria*	1:5	2.640	−0.550	5.828
	1:2	5.535	2.345	8.724
	1:1	7.702	4.512	10.891
	2:1	0.843	−2.346	4.032
	5:1	−9.628	−12.817	−6.438
*S. gregaria*	1:5	−23.895	−27.294	−20.494
	1:2	−17.878	−21.278	−14.478
	1:1	7.401	4.001	10.800
	2:1	−22.993	−26.392	−19.592
	5:1	−30.953	−34.353	−27.553
*S. exempta*	1:5	−41.504	−53.956	−29.052
	1:2	−35.560	−53.53	−17.585
	1:1	0.475	−11.977	12.927
	2:1	−6.064	−24.038	11.908
	5:1	−8.017	−20.470	4.434
*S. littoralis*	1:5	−23.037	−43.644	−2.430
	1:2	−9.955	−30.562	10.651
	1:1	−1.565	−22.171	19.042
	2:1	−13.086	−33.693	7.520
	5:1	−29.878	−50.484	−9.270

Note that ratios in which the angle $\beta$ overlaps zero implies no differences from CDO.

*Spodoptera exempta*, a diet specialist, displayed a somewhat similar nutrient array as *S. littoralis* for nutritional rails that were carbohydrate-biased relative to the intake target P:C ratio. However, for diets that were similar or protein-biased compared to the P:C ratio of the intake target, *S. exempta* displayed an imperfect resemblance to CDO. This mixture of responses showed in the plot of the angle $\beta$, in which values of $\beta$ progressively increased towards 90° as the P:C ratio increased (i.e., more protein) up until the nutritional rail with similar P:C ratio to the intake target, where the intake in the nutritional rail decreased (and hence, the angle $\beta$ was $\ge$ 90° (see Fig. 2f). Furthermore, contrary to the response observed in *S. littoralis*, the angle $\beta$ did not decrease as the nutritional rails became protein-biased, suggesting that *S. exempta* held the intake of imbalanced diets closer to the expected intake for CDO (see Fig. 2f, Table 2). Together, the results from the method suggest that *S. littoralis* can cope with surpluses of both carbohydrates and proteins equally well, whereas *S. exempta* can cope well with surplus of carbohydrate but tightly regulate the intake of nutrients in protein-biased diets.

## Discussion

Animals often need to regulate the intake of nutrients, a challenging task when animals feed on imbalanced diets. Rules evolved which enable animals to balance the costs and benefits of under- and over- consumption of nutrients in these situations (‘rules of compromise’) imposing important constrains on how animals eat^5,6^. Using the Geometric Framework for nutrition, studies have generated a rich collection of datasets that allow for these rules of compromise to be studied in details. However, the development of analytical methods for statistical inferences lagged behind^8,43,47^. In this study, I proposed an analytical method to study rules of compromise, which was validated using three landmark datasets of increasing complexity. This method provides two main contributions to the field, namely, (1) an intuitive framework for data visualisation of animal feeding and (2) a simple method to describe and test the rules of compromise in animal nutrition. This will help advance our understanding of how animals compromise the intake of nutrients when feeding in imbalanced diets.

Diet specialists are seemingly constrained in their ability to cope with overconsumption of nutrients and compromise on the intake of both nutrients tested (in this case, protein and carbohydrate), in accordance with the strategy of closest distance optimisation (CDO). This was evident in the array of *L. migratoria* and partly evident in the nutritional array of *S. exempta* (see Fig. 2c–f). CDO has been hypothesised as a general pattern in diet specialists, where the consumption of multiple nutrients are tightly regulated to ensure fitness^50,51^. Interestingly, in the cockroach *Blattella germanica*, in which some populations have evolved dietary specialisation to avoid glucose, rules of compromise did not comply with CDO (see^52^). Glucose-avoidance specialisation appears to be hardwired and individuals may be unable to compensate for underconsumption of nutrients via digestive processes^52^. One caveat is that^52^ only used three nutritional rails to construct the nutritional arrays and thus, the experimental design is not broad enough to allow for a proper test for CDO. Nonetheless, the model proposed here can be used in future studies to verify whether or not the nutrient arrays of diet specialists and generalists adhere to CDO rule of compromise.

The nutrient array of *D. melanogaster* was also constrained for overconsumption of imabalanced diets, particularly those with high carbohydrate, and resembled in many ways the array of a diet specialist even though *D. melanogaster* is widely defined as being diet generalist. Could the nutrient array be revealing that *D. melanogaster* is in truth a specialist species? This is unlikely, although not completely implausible scenario. The laboratory population used in the study by^14^ was inbred and could have behaved as a diet specialist. Moreover, a recent study using the Drosophila Genetic Reference Panel (DGRP) lines has shown considerable variability in *D. melanogaster* survival across different diets, in particular diets with high carbohydrate levels^53^. This aligns with the insights gained here using the Thales’ method: *Drosophila* nutrient array diverges more strongly from CDO as the concentration of carbohydrate (but not of protein) increases. Contrary to this, however, previous studies have shown that in high-sugar diets, flies have reduced responses to sweet taste which leads to overconsumption of the diet, a response that is mediated by the release of dopamine and the expression of the enzyme O-linked N-Acetylglucosamine transferase (OGT) in sweet-sensing neurons^54,55^. Moreover, sugar consumption is directly linked to female fecundity^56^ and male fertility is maximised at a relatively higher proportion of sugar consumption (compared with females)^14,19^. This highlights the importance of sugar-appetite to overall fitness. Thus, the seemingly divergent findings of the nutritional array studies and studies on the consumption of sugar in *Drosophila* literature remains subject of further molecular and physiological studies.

I have shown that the method proposed here can help the interpretation of more complex nutritional arrays which display mixed responses to nutrient imbalances. To be applied in empirical datasets, the method proposed here requires a GF choice experiment to determine the coordinates of the intake target, from which an accurate Thales’ circle can be drawn to analyse the nutritional array. It is also worth mentioning that the method presented here has some limitations. Firstly, I calculated the angle $\beta$ for individual datapoints in each nutritional rail relative to the average intake target. As a result, the estimates might suffer from uncertainty propagation (i.e. when the uncertainty in random variable are propagated when variables are combined). This is particularly important when variables are correlated, as failing to account for propagation of uncertainty can lead to underestimation of combined error. In the datasets used to validate the approach presented here, the errors associated with nutritional rails and intake targets were collected independently and are assumed to be uncorrelated, as most of the studies conducted in the field (e.g.^11,46^. In the datasets analysed here, the combined error is smaller than the individual errors in nutrient intake and thus, the Thales’ approach presented here shows a conservative estimate of statistical significance (Table S1). Recent studies have started to consider uncertainty propagation when measuring GF data within the context of the protein leverage hypothesis^57^ but this investigation lies beyond the scope of this study. Secondly, the Thales’ theorem applies in two, but not higher dimensions. This 2D-nutritional-arrays approach have been proposed as the best way to analyse rules of compromise^51^ and the method proposed in this study complies with this recommendation. Should the number of dimensions of the nutritional array increase, however, a new method has to be devised *or* the data has to be ‘sliced’ into lower dimension subsections (either by pairwise comparisons or through dimensionality reduction such as e.g., Principal Component Analysis), although such approach can transform the data in ways that might complicate analysis of the rules of compromise. High-dimensional data are considerably more difficult to interpret, and whether high-dimensional nutritional arrays are in themselves informative remains to be studied. So far, studies that investigated the rules of compromise have been of 2D, for which the method proposed here is perfectly suitable (e.g.,^12,14,15,19,21–23,28,41,43,44^, and others).

Using an ancient theorem known as the Thales’ theorem, I have developed an intuitive and reproducible analytical method to study the feeding patterns of animal in response to nutrient imbalances. This method advances our previous approaches and enables statistical analysis and interpretation of complex patterns in nutrient arrays. This opens up routes for the application of this method to the broader field of nutritional ecology, including recent translational studies using GF to investigate nutrition in health and (metabolic) diseases (e.g.^29,33,58^).

## Methods

### Datasets

I validated the method using three landmark datasets in the field of nutritional ecology:

1. The first was the data from^14^ on the nutritional responses in *Drosophila melanogaster*. This is a landmark paper because it was the first to demonstrate the nutritional trade-offs between lifespan, reproductive rate, and lifetime egg production (i.e., fitness), and that when given a choice, individuals feed on diets with nutrient ratios that maximise lifetime egg production. The experiments focused on the manipulation of the ratio of protein and carbohydrate (P:C ratio) of the diets. Flies were given seven P:C ratios (i.e., nutritional rails), namely, 0:1, 1:16, 1:8, 1:4, 1:2, 1:1, and 1.9^14^. This data has been extensively used for GF method development and thus, has gained a important status as a ground-truth in the field^38–40^.
2. The second dataset was for two locust species originally presented in Fig. 4 of^8^. The two species for which the nutrient array were extracted were *Locusta migratoria* (specialist, gregarious) and *Schistocerca gregaria* (generalist, solitary or gregarious). For the purpose of this paper, where I used the data for validation, I compared the nutrient arrays of both species but opted to omit the comparison for the solitary vs gregarious stage of *S. gregaria* presented in^8^. This is because my aim was not to replicate the original study, but to demonstrate the power of the method proposed here in identifying different shapes of nutrient arrays.This data also contained five nutritional rails with P:C ratios 7:35 (1:5), 14:28 (1:2), 21:21 (1:1), 28:14 (2:1) or 35:7(5:1).
3. The third dataset was for two Lepidopteran species of the *Spodoptera* genus: *S. littoralis* and *S. exempta*, the former a diet generalist and the latter, a diet specialist. Moths were given five P:C ratios (nutritional rails), namely, 35:7 (5:1), 28:14 (2:1), 21:21 (1:1), 14:28 (1:2) and 7:35 (1:5)^43,44^.

More details of the findings of the above studies can be found in the original publications.

### Statistical analyses

All analyses were conducted in R version 4.1.3^59^. Data handling was conducted using the tidyverse packages ‘dplyr 1.0-10’ and ‘tidyr 1.2.0’^60^. Data visualisation plots were done using the ‘ggplot2 3.4.0’ package^61^. I studied the stability of the probability distribution of errors on the estimates of the angle $\beta$ using a nutrient array with known angle (i.e., 90 °) and added error using the ‘rnorm’, ‘rpois’ and ‘rgamma’ functions in R, with increasing values of the the parameters related to the standard deviation of the distributions (i.e., ‘sd’, ‘lambda’, and ‘shape’ parameters, respectively). Simulation sample size was equal to *n* = 100. I ran 100 simulations, each with standard deviation of the data for each distribution increasing from 0.01 (virtually no error) to 100 (error equals the sample size) in steps of 0.5, totalling 59700 simulated observations of the angle estimates. I estimate the extent of the effects of increasing errors given the proportion of effect size over the error, represented by the proportion of error relative to the data (Fig. 1d). Next, I extracted average intake for each nutritional rail from^8^ manually using WebPlotDigitizer 4.2^62^. Errors in the intake of carbohydrate and protein for the dataset from^8^ were simulated from a normal distribution using the ‘rnorm’ function in R with the parameter ‘mean’ equal to the mean protein or carbohydrate observed in the data and standard deviation equals to 10. Because the errors were simulated, I did not apply test statistics to this dataset. This approach was necessary because the raw estimates of error was not available in the original dataset. In all datasets, the angle $\beta$ was estimated for each individual data point in the nutritional rails against the average coordinates of the intake target. This allowed me to estimate the 95% confidence intervals of the angle $\beta$, which were calculated using the ‘confint’ in-built function. R scripts are available in the Text S1 in the electronic supplementary material.

## Supplementary Information

Supplementary Information 1.

Supplementary Information 2.

Supplementary Information 3.

Supplementary Information 4.

Supplementary Information 5.

Supplementary Table S1.

## Data Availability

All data generated or analysed during this study are included in this published article and its supplementary information files. R script to reproduce the analysis is available in the electronic supplementary material.
