# Supplementary material for: Nutrigonometry IV: Thales’ theorem to measure the rules of dietary compromise in animals
Source: Sci Rep. 2023 May 8;13:7466. doi: 10.1038/s41598-023-34722-7 (PMC10167223; doi:10.1038/s41598-023-34722-7)
Supplement: Supplementary file 6 — Supplementary Table S1. [file 41598_2023_34722_MOESM6_ESM.pdf]

Table S1. Individual *versus* combined errors.

Species	Experiment	Nutrient	SD	SD (Combined)
Heliothis subflexa	Target	Carbohydrate	35.2	
	Rails		56.4	66.48
Heliothis virescens	Target		28.5	
	Rails		30.1	41.45
Heliothis subflexa	Target	Protein	33.6	
	Rails		41	53.00
Heliothis virescens	Target		38.5	
	Rails		43.8	58.31
Spodoptera exempta	Target	Carbohydrate	11.5	
	Rails		23.7	26.34
Spodoptera littoralis	Target		41.6	
	Rails		66	78.01
Spodoptera exempta	Target	Protein	13.7	
	Rails		16.4	21.36
Spodoptera littoralis	Target		58.8	
	Rails		68.3	90.12
